# Modulation of oxidative stress and metabolic burden in papillary thyroid cancer by SGLT2 and broad DPP inhibition

**DOI:** 10.3389/fmolb.2026.1875059

**Published:** 2026-07-29

**Authors:** Angelika Buczyńska-Backiel, Iwona Sidorkiewicz, Julia Redlińska, Maria Kościuszko, Agnieszka Adamska, Katarzyna Siewko, Anna Popławska-Kita, Adam Jacek Krętowski

**Affiliations:** 1 Clinical Research Centre, Medical University of Bialystok, Bialystok, Poland; 2 Clinical Research Support Centre, Medical University of Białystok, Bialystok, Poland; 3 Department of Gynecological Endocrinology and Adolescent Gynecology, Medical University of Białystok, Bialystok, Poland; 4 Department of Endocrinology, Diabetology and Internal Medicine, Medical University of Bialystok, Bialystok, Poland

**Keywords:** angioinvasion, iDPP, Isglt2, papillary thyroid cancer, thyroid cancer

## Abstract

**Introduction:**

Papillary thyroid cancer (PTC) exhibits metabolic reprogramming consistent with the Warburg effect, characterized by enhanced glycolysis, redox imbalance and dependence on anti-apoptotic mechanisms such as XIAP stabilization. Modulating glucose handling and oxidative status with SGLT2 or broad DPP inhibition may therefore reveal vulnerabilities within this metabolic–apoptotic axis. This study assessed their effects on oxidative stress, glucose-dependent metabolic activity and XIAP distribution and apoptosis-related signaling in thyroid-derived cell models.

**Methods:**

Two PTC lines (SCC147, MDA-T32) and a normal thyroid line (NTHY-ORI) were exposed for 48 h to an SGLT2 inhibitor (10^−7^ M), a broad, non-selective DPP inhibitor (iDPP) (10^−6^ M), or vandetanib (10^−6^ M), with doses selected by MTT viability profiling. Reactive oxygen species (ROS), lipid peroxidation (MDA), total antioxidative capacity (TAC), intracellular and extracellular XIAP, and glucose-normalized metabolic indices (IG, MS) were quantified.

**Results:**

Metabolic profiling showed that SGLT2 inhibition markedly increased IG and MS in SCC147 and NTHY-ORI, consistent with altered glucose-dependent metabolic responses. In contrast, in MDA-T32 SGLT2 inhibition lowered IG/MS values, indicating reduced metabolic activity and a distinct metabolic phenotype. Both inhibitors modulated oxidative stress in a cell-line–dependent manner. SGLT2 inhibition reduced ROS levels in SCC147 (p < 0.01), whereas broad DPP inhibition reduced ROS in NTHY-ORI (p < 0.05), with no significant change observed following SGLT2 inhibition in this cell line. A significant decrease in MDA following iSGLT2 treatment was observed in SCC147 cells, whereas iDPP significantly reduced MDA levels in NTHY-ORI cells. No significant changes were detected in MDA-T32 cells. TAC responses were cell-line dependent. iDPP increased TAC in MDA-T32 and SCC147 cells, whereas both vandetanib and iDPP reduced TAC in NTHY-ORI cells. iSGLT2 did not significantly affect TAC. Extracellular XIAP changes were heterogeneous across cell lines, with the highest levels observed under different conditions depending on the cellular model.

**Discussion:**

SGLT2 (10^−7^ M) and broad DPP inhibition (10^−6^ M) differentially modulated oxidative stress markers, glucose-associated metabolic indices and XIAP distribution in a strongly cell-line-dependent manner. SCC147 and NTHY-ORI exhibited marked changes in glucose-dependent metabolic indices following SGLT2 inhibition, whereas MDA-T32 showed lower glucose-dependent metabolic indices and a distinct metabolic phenotype. This divergence highlights distinct metabolic–redox vulnerabilities across thyroid cell types, underscoring the importance of cellular context in determining treatment response.

## Graphical Abstract

**Figure:**
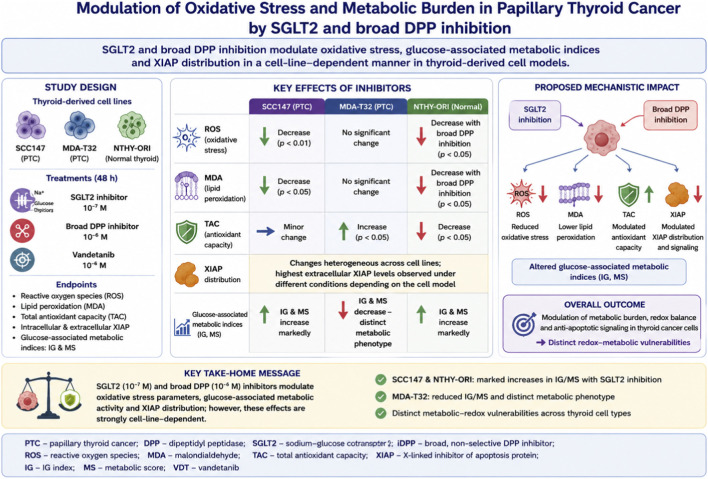

## 1 Introduction

Papillary thyroid cancer (PTC), the most common thyroid malignancy, is predominantly indolent, yet ∼10% of tumors display aggressive behavior, including angioinvasion, nodal or distant spread and persistent structural disease (Vigneri et al., 2015; Seib and Sosa, 2019; Lukas et al., 2013). Management of such cases remains burdensome: advanced tumors require total thyroidectomy and radioiodine (RAI), whereas de-escalation with lobectomy carries a measurable recurrence risk in larger or node-positive lesions (Ringel et al., 2025; Tronci et al., 2019). These clinical differences parallel distinct metabolic and redox phenotypes that shape iodide handling, oxidative-stress tolerance and treatment adaptability, indicating that metabolic features may refine risk stratification beyond standard clinical parameters (Buczyńska et al., 2023a; Buczyńska et al., 2023b; Buczyńska et al., 2021). Thus, PTCs with intensified glycolysis, elevated reactive oxygen species (ROS) or increased redox–metabolic activity exhibit more aggressive behavior, reduced RAI sensitivity and poorer outcomes (Cazarin et al., 2022; Kim et al., 2025; Erdian et al., 2025). Markers such as malondialdehyde (MDA) reflect lipid peroxidation and membrane instability, while increased glycolytic and pentose phosphate pathway flux enhances nicotinamide adenine dinucleotide phosphate (NADPH) production and redox buffering (Buczyńska et al., 2023c; Buczyńska et al., 2025; Kościuszko et al., 2023; Gatenby and Gillies, 2004; Hong et al., 2016; Marín-Hernández et al., 2011; Hsu and Sabatini, 2008). These adaptations promote apoptosis resistance, including stabilization of the X-linked inhibitor of apoptosis protein (XIAP), whereas extracellular XIAP denotes membrane damage and loss of viability (Cheung et al., 2020; Bertheloot et al., 2021; Obexer and Ausserlechner, 2014). Notably, no approved therapies directly target ROS, lipid oxidation, total antioxidative capacity (TAC) or XIAP-linked cytoprotection in PTC (Buczyńska et al., 2023a; Buczyńska et al., 2025; Buczyńska et al., 2022; Buczyńska et al., 2024a).

In this context, antidiabetic agents, particularly sodium–glucose cotransporter two inhibitors (SGLT2 inhibitors) and a broad dipeptidyl peptidase (DPP) inhibitors have gained attention as pharmacologically precise modulators of redox and metabolic homeostasis (Buczyńska et al., 2024a). SGLT2 inhibition reduces intracellular glucose influx independent of glucose transporters (GLUT) activity, limiting carbon entry into glycolysis and the tricarboxylic acid cycle (TCA), altering the nnicotinamide adenine dinucleotide (NADH/NAD^+^) redox couple, suppressing mitochondrial complex I–derived ROS, and attenuating oxidative modification of proteins and lipids (Wright, 2021; Basak et al., 2023; Lin and Tseng, 2014; Ali et al., 2023). SGLT2 inhibitors additionally activate AMP-activated protein kinase (AMPK) and the mechanistic target of rapamycin (mTOR), regulators of mitochondrial redox adaptation, glutathione synthesis and antioxidative gene expression (Xie et al., 2020). Broad DPP inhibition, via mechanisms independent of incretin biology, reduces NADPH oxidase 1/4–dependent reactive oxygen species (NOX1/NOX4-dependent ROS) and attenuates nuclear factor kappa-light-chain-enhancer of activated B cells (NF-κB)–mediated transcription of oxidative and inflammatory mediators, and modulates cell-surface proteolytic signalling that influences membrane integrity and apoptosis susceptibility (Gallwitz, 2019; Deacon, 2019; Ahrén, 2010). Both classes influence mitochondrial membrane potential, adenosine triphosphate (ATP) synthesis, glutathione turnover and the activity of enzymatic antioxidative, such as superoxide dismutase (SOD), glutathione peroxidase (GPx) and catalase providing pharmacologic leverage over cellular redox–metabolic systems without inducing genotoxicity (Chen et al., 2020; Ku et al., 2013; Lee et al., 2024). The choice of SGLT2 and DPP as pharmacological targets in this model is supported by evidence that both molecules are dysregulated in PTC. SGLT2 expression is elevated in PTC compared with normal thyroid tissue and correlates with proliferative markers (Buczyńska et al., 2024b). Experimental studies further show that SGLT2 inhibition reduces tumor growth, glucose uptake and glycolytic flux, while activating AMPK-dependent metabolic stress pathways (Wang et al., 2022). Likewise, DPP-4 is minimally expressed in normal thyroid but markedly upregulated in PTC, where its overexpression correlates with invasiveness, advanced stage and poorer prognosis (Wang et al., 2022; Gao et al., 2022), and functionally promotes migration through integrin- and FAK/AKT-mediated signaling (Gao et al., 2022; Polska et al., 2006). To our knowledge, previous studies in PTC have focused predominantly on selective DPP-4 inhibition, whereas the present study employed an experimental inhibitor with broader activity encompassing DPP-4 and DPP-8/9. Although this prevents attribution of the observed effects specifically to DPP-4, it provides a complementary perspective on how modulation of the broader DPP enzymatic network may influence redox and metabolic responses in thyroid-derived cells. Future studies will be required to dissect the relative contribution of individual DPP family members. Despite these mechanistic intersections, the coordinated effects of SGLT2 and DPP inhibition on oxidative stress and metabolic indices (IG, MS) and XIAP compartmentalization have not been characterized in PTC.

This study aimed to evaluate how SGLT2 and broad DPP inhibition affect oxidative stress markers, antioxidative capacity, glucose-dependent metabolic activity and XIAP distribution in normal and PTC-derived thyroid cell lines, in order to identify cell-line–specific differences in redox and metabolic responses relevant to aggressive PTC behavior.

## 2 Materials and methods

### 2.1 Cell lines and culture conditions

All experiments were performed at the Center for Experimental Medicine (CEM) under biosafety level-2 conditions. Cells were routinely monitored for morphology and viability and passaged before reaching full confluence. Three human thyroid-derived cell lines were used: two PTC lines, SCC147 (TPC-1 Human Papillary Thyroid Carcinoma Cell line, Merck) and MDA-T32 (CRL-3351, ATCC) and the non-tumoral thyroid follicular cell line NTHY-ORI (Nthy-ori 3-1 Cell Line human, 90011609-CDNA-20UL, Merck). Cells were cultured in RPMI-1640 medium supplemented with 10% fetal bovine serum (FBS) and 1% penicillin/streptomycin under standard conditions (37 °C, 5% CO_2_, humidified atmosphere). In all experiments, the cells were maintained in medium containing a reduced FBS concentration (2.5%). The reduced FBS concentration was applied to limit the influence of serum-derived growth factors that could interfere with metabolic and redox readouts. Prior to plating for the assays, cultures were gradually adapted to these conditions through five consecutive passages, ensuring that the cells reached a stable physiological state and minimizing adaptation-related variability.

#### 2.1.1 Reagents and inhibitors

The following pharmacological modulators were used: a selective SGLT2 inhibitor (iSGLT2), a non-selective DPP inhibitor (iDPP) and vandetanib (VDT), employed as a reference tyrosine-kinase inhibitor. Stock solutions were prepared in sterile phosphate-buffered saline (PBS) according to the manufacturer’s recommendations and diluted in culture medium immediately before use. Final working concentrations were established based on published pharmacokinetic data and MTT viability assays, in order to reflect clinically relevant exposure levels while avoiding cytotoxic effects and were as follows.

- iSGLT2: 1 × 10^−7^ M (SML2804, Dapagliflozin, Sigma-Aldrich, Darmstadt, Germany)
- iDPP: 1 × 10^−6^ M (broad DPP inhibition using a non-selective DPP inhibitor, 416200, Dipeptidyl peptidase IV inhibitor I, Sigma-Aldrich, Merck KGaA, Darmstadt, Germany)
- VDT: 1 × 10^−6^ M (SML2983, Vandetanib, Sigma-Aldrich, Merck KGaA, Darmstadt, Germany)

Importantly, the compound used in this study (DPP-IV-IN-2, CAS 136259-18-2) is a small-molecule experimental inhibitor (C_18_H_26_N_4_O_5_, MW 378.4 g/mol), commonly used in vitro studies. Unlike clinically approved an iDPP, it is not fully selective and inhibits both DPP-4 and DPP-8/9.

Vandetanib (VDT) inhibits Rearranged during Transfection (RET), Vascular Endothelial Growth Factor Receptor 2 (VEGFR2) and Epidermal Growth Factor Receptor (EGFR). Through suppression of the Phosphoinositide 3-kinase/Protein kinase B (PI3K/AKT) pathway and the Mitogen-activated protein kinase (MAPK) pathway (Puliafito et al., 2022). VDT was included as a clinically relevant reference condition because it represents an established targeted therapy used in advanced thyroid cancer. Since its mechanism of action differs fundamentally from those of SGLT2 and DPP inhibition, VDT was not intended to serve as a mechanistic control but rather as a contextual benchmark. Previous studies have shown that VDT may influence oxidative stress and mitochondrial function through inhibition of growth factor signaling pathways (Puliafito et al., 2022; Rodríguez-Hernández et al., 2020; Wells et al., 2010).

The negative control (KN) consisted of cells cultured in medium without inhibitors with addition of a vehicle volume (PBS) equivalent to that used for drug dilution.

#### 2.1.2 Cell viability assay (MTT)

To determine non-toxic, metabolically active concentrations of the inhibitors, preliminary viability assays were performed using the MTT method (MTT (3-(4,5-Dimethylthiazol-2-yl)-2,5-Diphenyltetrazolium Bromide, M6494, Invitrogen). This approach was chosen to investigate metabolic and redox modulation rather than cytotoxic or antiproliferative effects. Cells were seeded in 96-well plates at a density of 3000 cells/well and allowed to attach for 24 h. They were then exposed to a range of concentrations of iSGLT2, iDPP and VDT for 24 h and 48 h. Following incubation, MTT solution was added to each well and plates were incubated for the time recommended by the assay protocol. Formazan crystals were solubilized and absorbance was measured with a microplate reader. Concentrations that modified metabolic activity without inducing overt cytotoxicity were selected for subsequent experiments (iSGLT2 10^-7^ M, iDPP 10^−6^ M, VDT 10^−6^ M). The MTT assay was used to identify metabolically active, non-cytotoxic conditions rather than to determine dose–response relationships or IC_50_ values (Supplementary Material S1). The *in vitro* concentrations used in this study lie within the range of peak plasma levels reported for clinically used SGLT2 inhibitors (e.g., dapagliflozin) and DPP-4 inhibitors (e.g., sitagliptin) in patients with type 2 diabetes (Scheen, 2014; Que et al., 2023; Bennett, 2018; Szabadfi et al., 2014; Hayakawa et al., 2022). The MTT assay was used to exclude overt cytotoxic concentrations rather than to determine antiproliferative potency or IC_50_ values. Concentrations selected for further experiments were chosen to reflect clinically relevant exposure levels reported for SGLT2 and iDPP, allowing assessment of metabolic and redox effects under non-cytotoxic conditions.

#### 2.1.3 Experimental design and treatments

For the main experiments, cells were seeded in 6-well plates at 1 × 10^6^ cells/well in complete RPMI-1640 medium and allowed to adhere overnight. The following experimental conditions were then applied for 48 h.

- KN: control, no inhibitor (PBS only),
- iSGLT2: SGLT2 inhibitor 10^−7^ M,
- iDPP: a non-selective DPP inhibitor 10^−6^ M,
- VDT: vandetanib 10^−6^ M.

Final working concentrations were selected based on MTT viability assays to exclude cytotoxic conditions, and on published pharmacokinetic data to ensure clinically relevant exposure levels. At the end of the incubation, culture supernatants were collected, cleared by centrifugation to remove cell debris and stored at appropriate temperatures for subsequent analyses. Cells were washed with PBS, detached where required, and processed to obtain cell lysates for biochemical assays and protein quantification. All experiments were performed in at least three independent biological replicates. SGLT2 and DPP-4 protein levels were quantified in cell lysates using commercially available ELISA kits, according to the manufacturer’s instructions. Protein concentrations were normalized to total protein content. No significant differences in SGLT2 and DPP-4 protein levels were observed between treatment conditions in any of the analyzed cell lines (Supplementary Table S1).

### 2.2 Measurement of oxidative stress (ROS)

Intracellular ROS levels were quantified using the DCFDA (2′,7′-dichlorofluorescein diacetate) assay (ab113851, Abcam, Cambridge, United Kingdom). After 48 h of treatment, cells were incubated with DCFDA at a concentration recommended by the manufacturer for a defined period at 37 °C in the dark. The non-fluorescent DCFDA is de-esterified intracellularly and oxidized to fluorescent DCF in the presence of ROS. Fluorescence intensity was measured using a microplate reader (excitation/emission appropriate for DCF). Results are expressed as relative fluorescence units (RFU).

### 2.3 Lipid peroxidation (MDA)

Lipid peroxidation was assessed by determining malondialdehyde (MDA) levels in cell lysates. After 48 h of treatment, cells were harvested and lysed, and MDA was measured using a colorimetric or fluorometric assay based on the thiobarbituric acid reactive substance (TBARS) reaction, through which MDA can be quantified colorimetrically following its controlled reaction with thiobarbituric acid (TBARS Assay Kit; Cayman Chemical Corp., United States, 10009055), which was performed according to the manufacturer’s recommendations. Absorbance was measured with a microplate reader, and MDA concentrations were calculated from a standard curve.

### 2.4 Total oxidative and antioxidative capacity (TOC, TAC)

The total oxidative capacity (TOS) status was assessed using photometric immunodiagnostic assay (PerOx [TOS/TOC] kit, KC5100, 64625 Bensheim, Germany) and TAC status was determined by photometric assay (ImAnOx [TAS/TAC] Kit, KC5200, 64625 Bensheim, Germany). Briefly, for TOC the cumulative oxidative potential of the sample was quantified by oxidation of a probe to a colored or fluorescent product, whereas TAC reflected the ability of the sample antioxidative to inhibit oxidation of a standard substrate. Absorbance or fluorescence was measured with a microplate reader, and TOC/TAC values were normalized to protein concentration. TAC was used as an integrative index of antioxidative defense status under different treatment conditions.

### 2.5 XIAP quantification in cell lysates and culture media

XIAP concentrations were measured both in cell lysates and in culture supernatants to differentiate between intracellular maintenance of anti-apoptotic signaling and XIAP release associated with membrane damage (RAB0517-1KT, Sigma-Aldrich, Darmstadt, Germany) using enzyme-linked immunosorbent assay (ELISA). Following 48 h of treatment, cells were lysed using RIPA buffer containing protease inhibitors, and total protein concentrations were determined spectrophotometrically using a NanoDrop microvolume spectrophotometer (Thermo Fisher Scientific, United States), as described in Section 2.8. Absorbance was measured using a microplate reader and XIAP concentrations were calculated from standard curves supplied with the kit. Parallel measurements were performed in corresponding culture supernatants. XIAP concentrations were expressed as ng/mg protein for lysates and ng/mL for supernatants.

### 2.6 SGLT2 and DPP-4 quantification in cell lysates

SGLT2 and DPP-4 protein levels were quantified in cell lysates using ELISA. The following commercially available kits were used: Human DPP4 (CD26) ELISA Kit (ab252365, Abcam, Cambridge, United Kingdom) and Human SGLT2 (Sodium/glucose cotransporter 2) ELISA Kit (EH4455, Fine Test, Wuhan, China). Absorbance was measured using a microplate reader, and protein concentrations were calculated based on standard curves. The obtained values were normalized to total protein content.

### 2.7 Assessment of intracellular glucose content and calculation of metabolic indices

Intracellular glucose concentration was used as a normalized indicator of glucose availability, reflecting the balance between glucose influx and intracellular utilization. Intracellular glucose content was evaluated by measuring glucose concentration in cell lysates after 48 h of treatment. Glucose levels were quantified using an enzymatic colorimetric assay according to the manufacturer’s protocol. To account for differences in cell mass, glucose concentration was normalized to total protein content and expressed as G_norm (glucose per mg protein). Based on G_norm, two dimensionless metabolic indices were calculated:

- IG = –Z_G, where Z_G denotes the Z-score of G_norm across conditions within a given cell line;
- MS = 1/ln (G_norm).

Higher IG and MS values correspond to lower intracellular glucose levels and are interpreted as indicators of a relative increase in glucose-dependent metabolic activity. These indices were used as synthetic markers of metabolic activity and stress in each cell line and condition. This approach does not directly measure glucose uptake or consumption, as intracellular glucose levels reflect a dynamic balance between glucose influx, glycolytic utilization, and other metabolic pathways. Therefore, it should be interpreted as an indirect indicator of cellular metabolic state. It should be emphasized that intracellular glucose concentration does not directly reflect glucose uptake or consumption, but rather represents a steady-state balance between glucose influx and metabolic utilization. Therefore, the calculated indices (IG, MS) should be interpreted as indirect indicators of glucose-dependent metabolic state rather than direct measures of metabolic flux.

### 2.8 Protein quantification and result normalization

Total protein content in cell lysates was determined spectrophotometrically using a NanoDrop microvolume spectrophotometer (Thermo Fisher Scientific). After lysis in RIPA buffer containing protease inhibitors, samples were centrifuged to remove insoluble debris, and the supernatants were used for further analyses. For each lysate, 1–2 µL was loaded onto the NanoDrop pedestal and absorbance was recorded at 280 nm. Protein concentration was calculated using the instrument’s built-in protein quantification mode, with path length and extinction parameters set according to the manufacturer’s instructions. Protein concentration was used for initial normalization of oxidative stress and metabolic parameters rather than for absolute quantification. NanoDrop spectrophotometry was selected as a rapid and reproducible method minimizing sample handling and operator-dependent variability. Importantly, all samples were processed under identical conditions, which supports the validity of relative comparisons.

Further, all MTT, ROS, TOC, TAC and MDA results were normalized to control values (set as 100%). All XIAP values obtained from cell lysates were normalized to protein concentration (expressed as mg/mL) to allow comparison between experimental conditions and cell lines. Normalized intracellular glucose content (G_norm) per mg of protein prior to calculation of metabolic indices IG and MS.

### 2.9 Glucose measurement

Intracellular glucose concentration was measured in cell lysates using an enzymatic colorimetric assay based on the hexokinase/glucose-6-phosphate dehydrogenase (HK/G6PD) method on a Roche Cobas C111 analyzer (Roche Diagnostics, Basel, Switzerland; assay ID: 04657527190). In this assay, glucose is phosphorylated by hexokinase and subsequently oxidized, generating NADPH, which is quantified spectrophotometrically. The method determines glucose concentration and does not directly measure glucose uptake or consumption.

### 2.10 Statistical analysis

Due to non-normal distribution and small sample size, data were analyzed using non-parametric statistics. Comparisons between different cell lines were performed using the Kruskal–Wallis test followed by Dunn’s multiple comparison test with Holm–Sidak correction. Differences between treatment conditions (KN, VDT, iSGLT2, iDPP) within each cell line were analyzed using the Friedman test followed by Dunn’s multiple comparison test. Data within each cell line were treated as paired, as measurements were obtained from matched experimental replicates. A p-value <0.05 was considered statistically significant. Statistical analyses were performed using GraphPad Prism (GraphPad PRISM v. 9.5.1; GraphPad Software, Inc., San Diego, CA).

## 3 Results

### 3.1 Cell line-specific changes in glucose-dependent metabolic indices following SGLT2 and broad DPP inhibition

**FIGURE 1:**
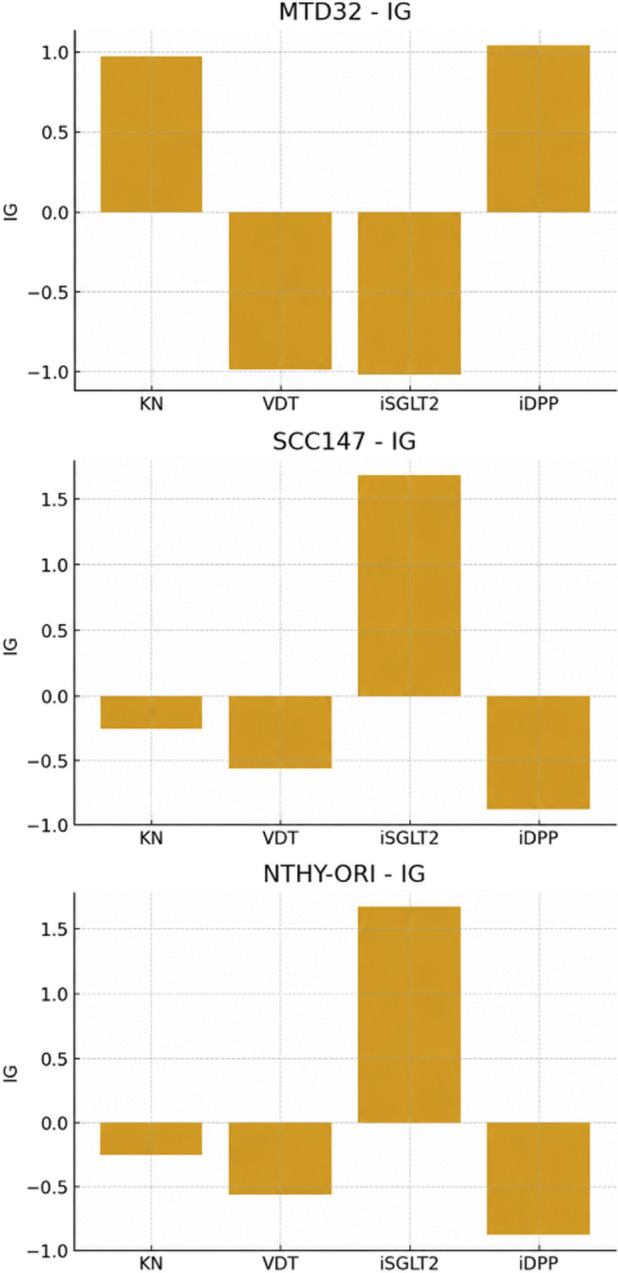
Metabolic indices (IG and MS) in thyroid cell lines under different treatment conditions. Metabolic indices IG and MS were calculated based on intracellular glucose content normalized to total protein (G_norm) in SCC147, MDA-T32, and NTHY-ORI cells after 48 h treatment with SGLT2 inhibitor (iSGLT2), Broad DPP inhibition using a non-selective DPP inhibitor (iDPP), and vandetanib (VDT). Higher IG and MS values may indicate a relative increase in glucose-dependent metabolic activity. Data are presented as mean ± SD of at least three independent biological replicates (n ≥ 3). Differences between treatment conditions within each cell line were analyzed using the Friedman test followed by Dunn’s multiple comparison test.

Higher IG and MS values may be consistent with increased glucose-dependent metabolic activity, whereas lower IG/MS values may reflect reduced metabolic demand and may reflect lower glucose-associated metabolic activity. Calculated metabolic activity differed substantially between the cell lines. In SCC147 and NTHY-ORI, SGLT2 inhibition produced the highest IG and MS values, indicating the highest glucose-dependent metabolic indices. DPP inhibition elicited a moderate rise in metabolic indices, whereas vandetanib generated the lowest IG/MS values, indicating lower glucose-dependent metabolic indices. In contrast, MDA-T32 displayed a distinct pattern: its highest metabolic indices occurred under control and iDPP-4–treated conditions, while SGLT2 inhibition markedly lowered IG/MS (Table 1; Figure 1). Notably, similar IG/MS patterns observed in SCC147 and NTHY-ORI reflect convergence at the level of glucose-normalized indices rather than identical raw metabolic behavior.

**TABLE 1:** Metabolism analysis.

Line	Condition	Gnorm (glucose/mg protein)	Z_G	IG = –Z_G	MS = 1/ln (Gnorm)
MDA-T32	KN	16.779	−0.967	0.967	0.355
MDA-T32	VDT	26.699	0.982	−0.982	0.304
MDA-T32	iSGLT2	26.882	1.018	−1.018	0.304
MDA-T32	DPP	16.444	−1.033	1.033	0.357
SCC147	KN	27.086	0.255	−0.255	0.303
SCC147	VDT	29.040	0.560	−0.560	0.297
SCC147	iSGLT2	14.614	−1.690	1.690	0.373
SCC147	DPP	31.063	0.875	−0.875	0.291
NTHY-ORI	KN	25.800	0.180	−0.180	0.309
NTHY-ORI	VDT	28.500	0.520	−0.520	0.298
NTHY-ORI	iSGLT2	16.200	−1.550	1.550	0.365
NTHY-ORI	DPP	29.800	0.850	−0.850	0.293

Normalized intracellular glucose content (G_norm, expressed as glucose per mg of protein), corresponding Z-scores (Z_G), and derived metabolic indices (IG, and MS) are presented for each cell line and experimental condition. IG, was calculated as the negative Z-score of G_norm (IG = –Z_G), while MS, was calculated as 1/ln (G_norm). Higher IG, and MS, values correspond to lower intracellular glucose levels and are interpreted as indicators of increased glucose-dependent metabolic activity. Conditions: KN, control (no inhibitor); VDT, vandetanib (10^−6^ M); iSGLT2 – SGLT2 inhibitor (10^−7^ M); DPP–a, non-selective DPP, inhibitor (iDPP) (10^−6^ M). Data represent mean values from independent biological replicates. Intracellular glucose concentration reflects the balance between glucose influx and intracellular utilization and does not constitute a direct measure of glucose uptake or consumption.

### 3.2 Differential modulation of intracellular ROS across thyroid-derived cell lines

**FIGURE 2:**
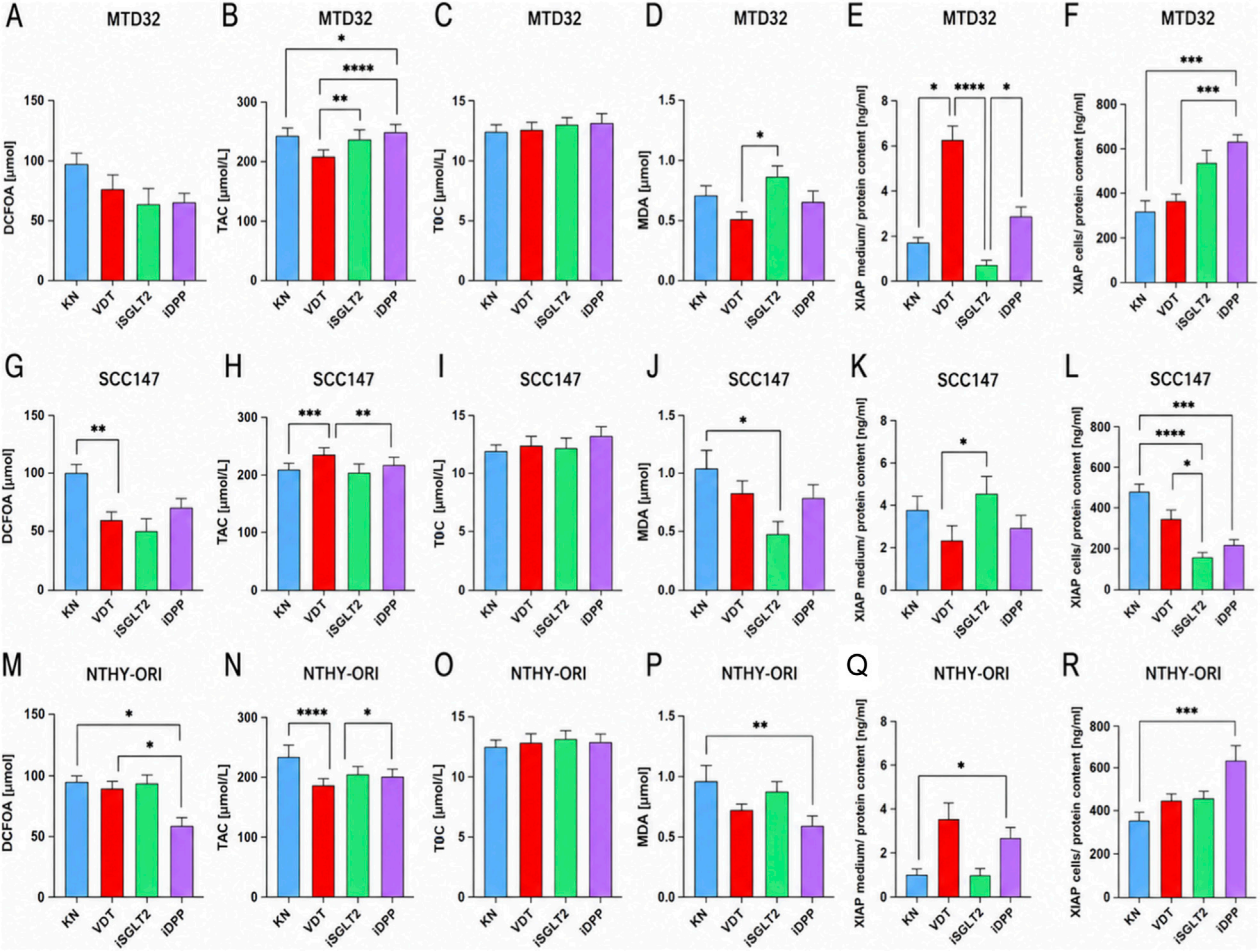
Effects of SGLT2 and DPP inhibition on oxidative stress and XIAP levels. **(A–F)** MTD32 cells: **(A)** intracellular reactive oxygen species (ROS; DCFDA), **(B)** total antioxidant capacity (TAC), **(C)** total oxidative capacity (TOC), **(D)** lipid peroxidation (MDA), **(E)** extracellular XIAP protein content, and **(F)** intracellular XIAP protein content. **(G–L)** SCC147 cells: **(G)** intracellular ROS (DCFDA), **(H)** total antioxidant capacity (TAC), **(I)** total oxidative capacity (TOC), **(J)** lipid peroxidation (MDA), **(K)** extracellular XIAP protein content, and **(L)** intracellular XIAP protein content. **(M–R)** NTHY-ORI cells: **(M)** intracellular ROS (DCFDA), **(N)** total antioxidant capacity (TAC), **(O)** total oxidative capacity (TOC), **(P)** lipid peroxidation (MDA), **(Q)** extracellular XIAP protein content, and **(R)** intracellular XIAP protein content. Cells were treated for 48 h with the SGLT2 inhibitor (iSGLT2), broad DPP inhibitor (iDPP), or vandetanib (VDT). Data are presented as mean ± SD from at least three independent biological replicates (n ≥ 3). Differences between treatment conditions within each cell line were analyzed using the Friedman test followed by Dunn’s multiple-comparison test. P < 0.05, P < 0.01, P < 0.001, P < 0.0001.

ROS responses differed between cell lines (Figures 2A,G,M). In MDA-T32 cells, neither iSGLT2 nor iDPP significantly altered ROS levels compared with untreated controls. In SCC147 cells, iSGLT2 significantly reduced ROS production (p = 0.0086), whereas iDPP produced only a non-significant trend toward lower values (p = 0.0525). In NTHY-ORI cells, ROS levels were significantly lower following iDPP treatment (p = 0.0461), while iSGLT2 had no significant effect. Furthermore, ROS values differed significantly between iSGLT2-and iDPP-treated NTHY-ORI cells (p = 0.0211) (Table 2). No statistically significant differences in TOC were detected among treatment groups in any of the investigated cell lines (Figures 2C,I,O).

**TABLE 2:** Markers analysis.

Parameter	Cell line	KN	VDT	iSGLT2	iDPP
ROS	MDA-T32	98.85 ± 19.79	x	51.72 ± 38.90 (p = 0.6109)	63.72 ± 24.15 (p = 0.1003)
	SCC147	100.63 ± 13.15	x	55.24 ± 22.83 (**p = 0.0086**)	81.26 ± 28.13 (p = 0.0525)
	NTHY-ORI	101.34 ± 19.56	x	101.04 ± 28.60 (p > 0.9999)	69.97 ± 21.64 (*p = 0.0461*)
TOC	MDA-T32	12.65 ± 1.11	12.84 ± 1.34 (p > 0.9999)	13.46 ± 1.04 (p = 0.6759)	13.81 ± 1.14 (p = 0.8336)
	SCC147	12.29 ± 0.89	12.87 ± 1.45 (p > 0.9999)	12.80 ± 1.68 (p > 0.9999)	13.39 ± 2.30 (p = 0.4316)
	NTHY-ORI	12.61 ± 0.77	13.15 ± 1.09 (p > 0.9999)	13.46 ± 1.81 (p > 0.9999)	12.42 ± 1.14 (p > 0.9999)
TAC	MDA-T32	255.50 ± 1.75	237.75 ± 3.80 (p = 0.4072)	259.87 ± 2.02 (p = 0.4194)	264.69 ± 3.83 (*p = 0.0194*)
	SCC147	208.72 ± 1.63	227.07 ± 12.40 (**p = 0.0003)	209.55 ± 3.07 (p > 0.9999)	215.80 ± 3.67 (*p = 0.0333*)
	NTHY-ORI	223.55 ± 10.66	199.95 ± 1.11 (***p < 0.0001)	207.50 ± 1.98 (p = 0.1912)	206.79 ± 9.99 (*p = 0.0479*)
MDA	MDA-T32	0.77 ± 0.16	0.61 ± 0.17 (p = 0.6384)	0.92 ± 0.24 (p > 0.9999)	0.68 ± 0.20 (p > 0.9999)
	SCC147	1.19 ± 0.45	0.95 ± 0.22 (p > 0.9999)	0.44 ± 0.25 (*p = 0.0182*)	0.74 ± 0.36 (p = 0.8088)
	NTHY-ORI	1.01 ± 0.30	0.71 ± 0.14 (p = 0.2669)	0.86 ± 0.14 (p > 0.9999)	0.56 ± 0.22 (**p = 0.0092**)
Intracellular XIAP	MDA-T32	312.03 ± 120.14	357.25 ± 76.30 (p > 0.9999)	546.20 ± 23.76 (*p = 0.0170*)	633.62 ± 71.96 (**p = 0.0002)
	SCC147	448.98 ± 80.45	334.45 ± 12.75 (p = 0.4716)	152.60 ± 86.06 (p < 0.0001)	207.06 ± 43.23 (p = 0.0006)
	NTHY-ORI	349.62 ± 77.29	456.46 ± 55.60 (p = 0.1980)	457.91 ± 74.65 (p = 0.2410)	638.50 ± 183.22 (p = 0.0008)
Extracellular XIAP	MDA-T32	1.72 ± 0.37	6.61 ± 2.02 (*p = 0.0239*)	0.55 ± 0.12 (p = 0.1977)	2.80 ± 1.26 (p > 0.9999)
	SCC147	2.96 ± 2.77	2.08 ± 0.80 (p > 0.9999)	4.28 ± 0.91 (p = 0.6032)	3.59 ± 1.65 (p > 0.9999)
	NTHY-ORI	1.19 ± 0.42	3.63 ± 2.47 (p = 0.0561)	1.29 ± 0.37 (p > 0.9999)	2.71 ± 0.38 (*p = 0.0362*)

Intracellular reactive oxygen species (ROS), total oxidative capacity (TOC), total antioxidative capacity (TAC), lipid peroxidation (MDA), and X-linked inhibitor of apoptosis protein (XIAP) levels in cell lysates (XIAP, cells) and culture media (XIAP, medium) are presented for each cell line and experimental condition. ROS, values are expressed as relative fluorescence units normalized to control conditions (set as 100%). TOC, and TAC are expressed in assay-specific units and normalized to protein content. MDA, concentrations reflect lipid peroxidation levels and are expressed in assay-specific units. XIAP, levels in cell lysates are normalized to total protein content (ng/mg protein), while XIAP, levels in culture media are expressed as ng/mL. Conditions: KN, control (no inhibitor); VDT, vandetanib (10^−6^ M); iSGLT2 – SGLT2 inhibitor (10^−7^ M); iDPP–a, non-selective DPP, inhibitor (10^−6^ M). Data are presented as mean ± standard deviation (SD) from independent biological replicates (n ≥ 3). Symbol “x” indicates that the measurement was not performed for this condition (significant values (p < 0.05) were bolded).

### 3.3 Cell line-dependent effects on lipid peroxidation

Changes in lipid peroxidation were heterogeneous across cell lines (Figures 2D,J,P). In MDA-T32 cells, iSGLT2 treatment was associated with numerically higher MDA levels, although the difference versus control was not statistically significant. The only significant difference in this cell line was observed between vandetanib and iSGLT2 (p = 0.0169).

In SCC147 cells, iSGLT2 significantly reduced MDA concentrations compared with untreated controls (p = 0.0182), whereas iDPP did not significantly affect MDA levels.

In NTHY-ORI cells, iDPP significantly decreased MDA concentrations (p = 0.0092), while neither vandetanib nor iSGLT2 significantly altered lipid peroxidation (Table 2).

### 3.4 Distinct antioxidative responses among thyroid-derived cell lines

Total antioxidant capacity exhibited cell-line-dependent responses (Figures 2B,H,N). In MDA-T32 cells, iDPP significantly increased TAC compared with controls (p = 0.0194), whereas neither vandetanib nor iSGLT2 significantly affected TAC values. Additional differences were observed between vandetanib and iSGLT2 (p = 0.0016) and between vandetanib and iDPP (p < 0.0001).

In SCC147 cells, TAC values were significantly higher following vandetanib treatment (p = 0.0003) and iDPP treatment (p = 0.0333), whereas iSGLT2 did not significantly modify TAC. A significant difference was also observed between vandetanib and iSGLT2 (p = 0.0032).

In NTHY-ORI cells, TAC was significantly lower after vandetanib treatment (p < 0.0001) and after iDPP treatment (p = 0.0479), while iSGLT2 did not induce significant changes. A difference was additionally observed between vandetanib and iSGLT2 (p = 0.0479) (Table 2).

### 3.5 Heterogeneous XIAP responses following SGLT2 and broad DPP inhibition

#### 3.5.1 Cell line-specific intracellular XIAP responses

SGLT2 Intracellular XIAP responses were strongly cell-line dependent (Figures 2F,L,S). In MDA-T32 cells, both iSGLT2 (p = 0.0170) and iDPP (p = 0.0002) significantly increased intracellular XIAP concentrations compared with untreated controls. In addition, intracellular XIAP levels differed significantly between vandetanib and iDPP-stimulated cells (p = 0.0009).

In SCC147 cells, both iSGLT2 (p < 0.0001) and iDPP (p = 0.0006) significantly reduced intracellular XIAP concentrations relative to controls. A significant difference was also observed between vandetanib and iSGLT2 (p = 0.0284).

In NTHY-ORI cells, iDPP significantly increased intracellular XIAP concentrations (p = 0.0008), whereas the increase observed after iSGLT2 treatment did not reach statistical significance (Table 2).

#### 3.5.2 Heterogeneous extracellular XIAP release patterns

Extracellular XIAP responses varied substantially between cell lines (Figures 2E,K,R). In MDA-T32 cells, extracellular XIAP concentrations were significantly increased following vandetanib treatment compared with controls (p = 0.0239). A marked difference was also observed between vandetanib and iSGLT2 (p < 0.0001), as well as between iSGLT2 and iDPP (p = 0.0119).

In SCC147 cells, no significant differences versus controls were detected. Although extracellular XIAP values were numerically higher following iSGLT2 treatment, this increase did not reach statistical significance. A significant difference was observed only between vandetanib and iSGLT2 (p = 0.0303).

In NTHY-ORI cells, extracellular XIAP concentrations were significantly increased after iDPP treatment compared with controls (p = 0.0362), whereas iSGLT2 did not significantly affect extracellular XIAP levels (Table 2; Figure 2). Overall, changes in extracellular XIAP were heterogeneous and cell-line dependent, with no consistent reduction observed across all conditions.

## 4 Discussion

Given that DPP-4 is markedly overexpressed in PTC and its expression correlates with tumor aggressiveness and poorer clinical outcomes, modulation of the DPP enzymatic system has emerged as a biologically relevant strategy in thyroid cancer. Lee et al. demonstrated that DPP-4 expression was associated with unfavorable clinicopathological features in PTC and showed that both DPP-4 silencing and pharmacological inhibition attenuated malignant characteristics in experimental models (He et al., 2020). In this context, the present study investigated whether modulation of redox balance, antioxidative capacity, glucose-associated metabolic state, and XIAP distribution could also be influenced by inhibition of the broader DPP enzymatic network. Importantly, because the inhibitor used in our experiments is not selective and targets DPP-8/9 in addition to DPP-4, the observed effects should not be interpreted as being specific to DPP-4 alone (Lee et al., 2017). The present study investigated whether SGLT2 and broad DPP inhibition modulate oxidative stress, antioxidative capacity, glucose-dependent metabolic activity, and XIAP distribution in thyroid-derived cell models. Importantly, the present study was not designed to evaluate cytotoxic or antiproliferative effects of the investigated inhibitors. Instead, we focused on their ability to modulate cellular metabolism and redox balance under physiologically relevant conditions. For this reason, concentrations were selected within clinically achievable ranges, and the MTT assay was used solely to confirm the absence of overt cytotoxicity. Metabolic modulation emerged as an early and robust effect. SGLT2 inhibition increased glucose-dependent metabolic indices (IG, MS) in SCC147 and NTHY-ORI, but decreased glucose-dependent metabolic indices in MDA-T32, indicating that SGLT2-dependent glucose handling is highly cell-line-specific. Similar context-dependent responses have been described in cardiac models, where SGLT2 inhibitors remodel mitochondrial metabolism and attenuate oxidative overload through AMPK activation, although the magnitude and direction of these effects vary among cell types. Consistently, Koizumi et al. and Peng et al. demonstrated that dapagliflozin mitigates high-glucose-induced metabolic overload by suppressing NADPH-oxidase-derived ROS and restoring antioxidative enzyme activity (Peng et al., 2024; Koizumi et al., 2023).

These observations align with our findings, where we observed pronounced context-dependent responses, underscoring the importance of avoiding generalized interpretations when describing redox–metabolic effects in thyroid cancer models. SGLT2 inhibition produced the most pronounced overall redox modulation, although the direction and magnitude of changes differed between cell lines. This pattern accords with studies showing that SGLT2 inhibitors attenuate mitochondrial ROS production, enhance AMPK signaling and activate the nuclear factor erythroid 2-related factor 2 (NRF2) antioxidative axis (Yaribeygi et al., 2023; Goedeke et al., 2024). Guerrero-Mauvecin et al. reported upregulation of SOD, GPx and catalase with SGLT2 inhibition (Guerre et al., 2025), while Llorens-Cebrià et al. demonstrated improved renal redox balance and mitochondrial homeostasis under empagliflozin (Llorens-Cebrià et al., 2022). In the present study, broad DPP inhibition produced a milder but directionally similar redox effect. Moderate reductions in ROS and MDA, together with cell-line–dependent changes in TAC—particularly in SCC147 and NTHY-ORI—align with reports showing that sitagliptin lowers oxidative burden through NOX inhibition and suppression of NF-κB (Ramos et al., 2022). Importantly, several independent studies indicate that DPP-4 inhibitors can restore NRF2-driven antioxidative signaling via TXNIP- and PKC-dependent mechanisms. Although NRF2 activation was not directly evaluated here, the overall pattern of redox-related changes observed in our models is compatible with mechanisms previously linked to NRF2 activation. This suggests that the antioxidative effects detected in our assays fit within the broader mechanistic framework described in the literature, where both SGLT2 and non-selective DPP inhibition potentially converge on pathways known to enhance NRF2-mediated cytoprotection (Bao et al., 2021; Ciavardelli et al., 2017). These mechanisms may align with the heterogeneous redox responses observed in our models, particularly in non-tumoral cells.

These contrasting profiles illustrate why PTC-derived cell lines may respond differently to glucose-modulating interventions. Importantly, the relationship between glucose-dependent metabolic activity, oxidative stress, and XIAP distribution differed markedly between the analyzed cell lines. SGLT2 inhibition produced a bidirectional metabolic response: SCC147 and NTHY-ORI increased their glucose-dependent metabolic indices (based on IG, MS), whereas MDA-T32 exhibited lower glucose-associated metabolic indices, indicating a distinct response to SGLT2 inhibition. This pattern suggests that responses to SGLT2 inhibition are shaped more by intrinsic metabolic wiring than by histologic aggressiveness alone. Although SCC147 and MDA-T32 are both invasive PTC models, they appear to engage distinct metabolic defense strategies: SCC147 appears to enhance glucose-dependent metabolic compensation when transport is perturbed, whereas MDA-T32 appears to downscale this component of metabolism, possibly reflecting a distinct adaptive response to metabolic stress (Adekola et al., 2012; Cunha et al., 2023). At the molecular level, SCC147 (TPC1-derived) and MDA-T32 represent genetically distinct PTC models with well-characterized metabolic differences, which were reflected in our experimental readouts. In line with previous descriptions of TPC1-based systems, SCC147 in our study displayed a pronounced glucose-associated metabolic phenotype and showed a marked increase in glucose-dependent metabolic indices following SGLT2 inhibition. This behavior is consistent with the elevated glycolytic and pentose phosphate pathway activity and the higher expression of glucose-handling enzymes such as pyruvate kinase M2 (PKM2) and G6PD reported for TPC1-derived lines relative to NTHY-ORI. MDA-T32, despite harboring the BRAFV600E mutation typically associated with GLUT1 upregulation and enhanced glycolysis in PTC, exhibited a contrasting pattern in our experiments. Instead of further increasing glucose-associated metabolic indices under SGLT2 inhibition, MDA-T32 exhibited lower glucose-associated metabolic indices, suggesting a distinct response to SGLT2 inhibition. This inverse response suggests that, in our model, BRAFV600E-associated signaling may coexist with stress-induced metabolic rerouting toward a more oxidative or hybrid phenotype, consistent with reports describing enhanced reliance on OXPHOS in aggressive PTC subtypes. NTHY-ORI, which maintains physiological coupling between iodide-handling machinery and glucose-transporter regulation, showed a modest and buffered metabolic response to both inhibitors in our dataset. This aligns with its status as a non-malignant reference line with preserved homeostatic regulation of glucose utilization. (Lee et al., 2023; Nagayama and Hamada, 2022; Suh et al., 2019; Ruan et al., 2015). Such heterogeneity may further reflect differences in metabolic flexibility and the ability of individual cell lines to adapt to glucose restriction. Rather than producing a uniform metabolic response, cancer cells can prioritize energy homeostasis and redox stability by dynamically adjusting substrate utilization and ATP demand. In this context, the increase in IG/MS observed in SCC147 and NTHY-ORI may represent an adaptive response aimed at maintaining energy production under reduced glucose availability. In contrast, the reduction in IG/MS in MDA-T32 suggests a distinct strategy, in which metabolic activity is downregulated to limit energy expenditure and prevent excessive oxidative stress. Such divergence indicates that cellular responses to SGLT2 inhibition are not solely determined by baseline glycolytic phenotype, but also by the capacity to balance metabolic demand with redox and energetic constraints under stress conditions (Irokawa et al., 2021; Kumar and Bamezai, 2015; Kurashige et al., 2023). Our findings are also compatible with the broader concept that oncogenic background does not enforce a single metabolic phenotype in thyroid cancer. Although BRAFV600E is commonly associated with increased glucose uptake and suppression of oxidative phosphorylation, thyroid tumors and cell models carrying this alteration may still retain substantial metabolic plasticity and switch toward hybrid or mitochondria-supported states depending on stress conditions. In thyroid systems, BRAF-driven signaling has been linked to increased glucose uptake, reduced oxygen consumption, and enhanced aerobic glycolysis, whereas other studies indicate that more aggressive thyroid phenotypes can simultaneously rely on mitochondrial adaptation, autophagy-supported respiration, or altered OXPHOS integrity. In this context, the divergent response of MDA-T32 in our study may reflect stress-induced metabolic rerouting rather than a contradiction of the glycolytic role of BRAFV600E (Lee et al., 2011; Tsybrovskyy et al., 2022; Díaz-Gago et al., 2024).

Importantly, our data suggest that metabolic adaptation, oxidative stress control, and XIAP distribution should be interpreted as an interconnected response rather than as isolated readouts. In SCC147, the metabolic shift observed after SGLT2 inhibition was accompanied by lower ROS and MDA, consistent with the idea that coordinated changes in glucose-associated metabolic indices may accompany alterations in redox status. By contrast, in MDA-T32, lower glucose-associated metabolic indices were not accompanied by a parallel increase in oxidative damage, suggesting that this model may rely on alternative mechanisms to maintain redox stability. This interpretation is biologically plausible because redox-regulated changes in glycolytic enzymes such as PKM2 can divert metabolic flux toward NADPH-producing pathways, thereby limiting oxidative stress, while BRAF-driven thyroid cancer models may engage distinct mitochondrial adaptations under metabolic pressure (Kumar and Bamezai, 2015; Lee et al., 2011). The downstream consequences of these metabolic and redox effects were reflected in XIAP distribution. SGLT2 inhibition significantly increased intracellular XIAP in MDA-T32 cells, whereas the increase observed in NTHY-ORI did not reach statistical significance, whereas SCC147 cells did not show a comparable increase, suggesting altered regulation of cytoprotective signaling in a cell-line–dependent manner, potentially reflecting differences in mitochondrial stability and apoptosis-related processes. This pattern is consistent with the antioxidative and mitochondrial-stabilizing effects described by Koizumi et al. and Peng et al., who showed that reduced mitochondrial ROS and AMPK-driven metabolic rebalancing suppress caspase activity and promote XIAP retention (Peng et al., 2024; Koizumi et al., 2023). NRF2 activation, reported by Nguyen et al., further supports increased XIAP stability through its role in maintaining anti-apoptotic protein homeostasis (Nguyen et al., 2003). Broad DPP inhibition also modified XIAP distribution, although the direction and magnitude of changes varied among cell lines, aligning with its milder redox–metabolic influence. Variable intracellular and extracellular XIAP responses observed in our study are consistent with the heterogeneous redox–metabolic effects described previously (Ramos et al., 2022; Cao et al., 2021). The antioxidative effects reported by Ramos et al. also correspond with partial XIAP stabilization in our model (Ramos et al., 2022). Clinically, XIAP overexpression is linked to more aggressive PTC behavior. Elevated XIAP levels correlate with older age, larger tumor size, lymph node metastasis and shorter disease-free survival (Hussain et al., 2015; Yim et al., 2011; Gao et al., 2019) and broadly across cancers XIAP is associated with poorer overall and disease-free survival (Gao et al., 2019; Xiao et al., 2007). Thus, the increase in intracellular XIAP observed with SGLT2 and broad DPP inhibition may reflect altered apoptosis-related signaling; however, because extracellular XIAP responses were heterogeneous and membrane integrity was not directly assessed, these findings cannot be unequivocally interpreted as evidence of cytoprotection. These findings underscore the need to determine whether pharmacological XIAP stabilization in thyroid cancer enhances tolerance to oxidative stress or inadvertently reinforces survival signaling, particularly in aggressive or RAI-refractory disease. Within the limitations of the present study, the observed XIAP redistribution should be interpreted cautiously and cannot distinguish between adaptive responses and injury-related processes. In SCC147 cells, the combination of decreased intracellular XIAP and increased extracellular XIAP following SGLT2 inhibition may instead reflect enhanced cellular stress or membrane instability rather than a protective response.

This study has several limitations. First, all experiments were performed in two-dimensional monolayer cultures using a limited panel of thyroid-derived cell lines, which may not fully represent the metabolic and redox heterogeneity of patient tumors. Second, we did not quantify SGLT2 or DPP activity, mitochondrial membrane potential or the activity of specific antioxidative enzymes, preventing a direct mechanistic link between inhibitor exposure and subcellular targets. Because transporter abundance was not measured, functional responses cannot be directly attributed to baseline SGLT2 or DPP-4 levels. The lack of changes in SGLT2 and DPP-4 protein levels across conditions supports the notion that the observed effects are driven by functional inhibition rather than altered protein expression. The limited impact on cell viability, particularly in SCC147 cells, is therefore consistent with the intended experimental design and supports the interpretation that the observed effects reflect metabolic modulation rather than cell death. Determination of IC_50_ values was not performed, as this parameter is primarily relevant for cytotoxic or antiproliferative studies and was not aligned with the objective of the present work. This approach is consistent with the known pharmacological profiles of SGLT2 and iDPP, which are not primarily cytotoxic agents but rather modulators of metabolic and redox pathways. The inhibitor used in this study is not fully selective for DPP-4 and may also affect DPP-8 and DPP-9. Therefore, the observed effects cannot be attributed exclusively to DPP-4 inhibition and should be interpreted as resulting from broader DPP inhibition. Future studies employing selective DPP-4 inhibitors, selective DPP-8/9 inhibition, or genetic approaches such as gene silencing will be required to determine the relative contribution of individual DPP family members to the observed redox and metabolic effects. Moreover, IG and MS do not differentiate between glycolysis, oxidative phosphorylation (OXPHOS), or pentose phosphate pathway (PPP) activity. Thus, the assessment of intracellular glucose content does not allow discrimination between glucose uptake and downstream metabolic utilization. A major limitation of this study is that glucose metabolism was not assessed using direct methods such as glucose uptake assays or metabolic flux analysis. Instead, intracellular glucose concentration was measured, which reflects the balance between glucose influx and utilization rather than glucose consumption *per se*. Therefore, the metabolic indices derived from these measurements should be interpreted with caution and considered as indirect markers. Importantly, the primary focus of this study was on oxidative stress and redox balance, and glucose-related parameters were included as supportive, exploratory measures rather than primary endpoints. The reduced glucose-dependent metabolic indices observed in MDA-T32 cells may reflect a shift toward alternative energy pathways, potentially involving increased mitochondrial activity. However, this interpretation remains hypothetical, as mitochondrial function was not directly assessed in the present study. Future studies should include direct evaluation of mitochondrial activity and ROS origin, for example, using MitoSOX-based assays or metabolic flux analysis. Future studies should also incorporate direct glucose uptake assays (e.g., 2-NBDG) and metabolic flux analysis (e.g., Seahorse) to better characterize cellular bioenergetics. Third, XIAP measurements were limited to total intra- and extracellular levels without evaluating caspase activity or downstream apoptotic signaling. Therefore, the present study does not allow discrimination between adaptive XIAP redistribution and injury-related membrane damage. Although NanoDrop-based protein quantification is less precise for absolute measurements, it provided consistent normalization across identically processed samples; however, future studies may incorporate complementary methods such as BCA or Bradford assays to improve quantitative accuracy. Finally, the study did not assess long-term survival, radioiodine handling or interactions with current systemic therapies. Future studies should therefore use three-dimensional or *in vivo* models, include systematic profiling of transporters and antioxidative enzymes, and test whether pharmacological modulation of SGLT2 or DPP can synergize with radioiodine, tyrosine kinase inhibitors or XIAP-targeted approaches in aggressive PTC. Despite these limitations, our data demonstrate that SGLT2 and broad DPP inhibition modulate oxidative stress parameters, glucose-associated metabolic indices and XIAP distribution in a cell-line-dependent manner. SGLT2 inhibition exerted the strongest integrated effects across markers, while both inhibitors modulated XIAP distribution in a cell-line-dependent manner. Although these agents are not anticancer therapies for PTC, they uncover redox–metabolic vulnerabilities that could inform complementary therapeutic strategies.

## 5 Conclusion

This study demonstrates that pharmacological inhibition of SGLT2 (10^−7^ M) and broad DPP inhibition (10^−6^ M) exerts distinct redox–metabolic effects in PTC cell lines (SCC147 and MDA-T32) and in the non-tumoral thyroid line NTHY-ORI. The magnitude and direction of these responses were strongly cell-line dependent. SGLT2 inhibition significantly reduced ROS levels in SCC147 cells and decreased lipid peroxidation in SCC147, whereas broad DPP inhibition reduced ROS and MDA levels in NTHY-ORI cells. Total antioxidant capacity responses differed across cell lines, with significant changes observed following broad DPP inhibition in MDA-T32 and SCC147 cells and following both vandetanib and broad DPP inhibition in NTHY-ORI cells.

SGLT2 inhibition markedly altered glucose-associated metabolic indices (IG and MS), increasing these indices in SCC147 and NTHY-ORI while decreasing them in MDA-T32, indicating distinct glucose-associated metabolic phenotypes among thyroid-derived cells. Because these indices are derived from steady-state intracellular glucose concentrations, they should be interpreted as indirect indicators requiring further validation with dedicated metabolic assays.

Both inhibitors also modulated intracellular and extracellular XIAP distribution in a cell-line-dependent manner, without a consistent pattern across all models. Intracellular XIAP increased in MDA-T32 following both treatments, decreased in SCC147, and increased in NTHY-ORI primarily after broad DPP inhibition, highlighting heterogeneous apoptosis-related responses.

Collectively, our findings indicate that SGLT2 and broad DPP inhibition modulate oxidative stress parameters, glucose-associated metabolic indices, and XIAP distribution in thyroid-derived cell models without inducing overt cytotoxicity. Although these compounds are not intended as anticancer agents in PTC, the observed heterogeneity of responses reveals distinct redox–metabolic vulnerabilities and supports further investigation of redox–metabolic modulation as a complementary biological strategy alongside established therapeutic approaches.

## Data Availability

The datasets analyzed during the current study are available from the corresponding author on reasonable request.
