# Supplementary material for: Modulation of oxidative stress and metabolic burden in papillary thyroid cancer by SGLT2 and broad DPP inhibition
Source: Front Mol Biosci. 2026 Jul 29;13:1875059. doi: 10.3389/fmolb.2026.1875059 (PMC13461471; doi:10.3389/fmolb.2026.1875059)
Supplement: Supplementary file 1 [file DataSheet1.docx]

**Supplementary Material 1.** **Cell viability (MTT assay) after inhibitor exposure.**
Cell viability was assessed using the MTT assay after 24 h and 48 h exposure to SGLT2 inhibitor (iSGLT2), broad DPP inhibitor (iDPP), and vandetanib (VDT) in SCC147, MDA-T32, and NTHY-ORI cell lines. Data are presented as percentage of control (NC = 100%). The assay was used to exclude overt cytotoxic concentrations and to select metabolically active, non-cytotoxic conditions for subsequent analyses. *p < 0.05, **p < 0.01, ***p < 0.001, **** p<0.0001 vs. control (NC). Data are presented as mean ± SD of at least three independent biological replicates (n ≥ 3). Differences between treatment conditions within each cell line were analyzed using the Friedman test followed by Dunn’s multiple comparison test.

**Supplementary Table 1.** Normalized DPP-4 and SGLT2 protein levels (ELISA, lysates)

| CELL LINE | KN | VDT | iSGLT2 | iDPP |
| --- | --- | --- | --- | --- |
| DPP-4 | | | | |
| MDA-T32 | 10.4 | 10.8 | 10.9 | 10.2 |
| SCC147 | 11.7 | 11.1 | 11.9 | 11.4 |
| NTHY-ORI | 2.17 | 2.25 | 2.30 | 2.20 |
| SGLT2 | | | | |
| MDA-T32 | 9.2 | 9.1 | 9.3 | 9.15 |
| SCC147 | 8.2 | 8.0 | 8.3 | 8.1 |
| NTHY-ORI | 7.9 | 8.1 | 7.8 | 8.0 |

DPP-4 and SGLT2 protein levels were quantified in cell lysates using enzyme-linked immunosorbent assay (ELISA) and normalized to total protein content. Values are presented for each cell line and experimental condition. Conditions: KN – control (no inhibitor); VDT – vandetanib (10⁻⁶ M); iSGLT2 – SGLT2 inhibitor (10⁻⁷ M); iDPP – Broad DPP inhibition using a non-selective DPP inhibitor (10⁻⁶ M). No significant differences in DPP-4 or SGLT2 protein levels were observed between treatment conditions within any of the analyzed cell lines, indicating that the applied inhibitors did not affect protein abundance under the experimental conditions.
